# Supplementary material for: Harnessing Quantum Capacitance in 2D Material/Molecular Layer Junctions for Novel Electronic Device Functionality
Source: Nanomaterials (Basel). 2024 Jun 3;14(11):972. doi: 10.3390/nano14110972 (PMC11173504; doi:10.3390/nano14110972)
Supplement: Supplementary file 1 [file nanomaterials-14-00972-s001.zip › nanomaterials-3014215-supplementary.pdf]

Electronic Supplementary Information

(ESI)

Harnessing Quantum Capacitance in 2D Material/Molecular Layer Junctions for Novel Electronic Device Functionality

Bhartendu Papnai [1,2,3], Ding-Rui Chen [4,5], Rapti Ghosh [6,7], Zhi-Long Yen [4,8,9], Yu-Xiang Chen [4,8,9], Khalil Ur Rehman [4,8,9], Hsin-Yi Tiffany Chen [1,10,11], Ya-Ping Hsieh [4] and Mario Hofmann [3,*]

[1] Department of Engineering and System Science, National Tsing Hua University, Hsinchu City 300044, Taiwan; bharatpapnai@gmail.com (B.P.); hsinyi.tiffany.chen@gapp.nthu.edu (H.-Y.T.C.)

[2] Nanoscience and Technology Program, Taiwan International Graduate Program, Academia Sinica, Taipei 106, Taiwan

[3] Department of Physics, National Taiwan University, Taipei 10617, Taiwan

[4] Institute of Atomic and Molecular Sciences, Academia Sinica, Taipei, 10617, Taiwan; dingruichen54@gmail.com (D.-R.C.); d12551007@ntu.edu.tw (Z.-L.Y.); d11551008@ntu.edu.tw (Y.-X.C.); khalilmrwt@gmail.com (K.U.R.); yphsieh@gate.sinica.edu.tw (Y.-P.H.)

[5] Department of Electrical Engineering and Computer Sciences, Massachusetts Institute of Technology, Cambridge, MA 02139, USA

[6] Pritzker School of Molecular Engineering, University of Chicago, Chicago, IL 60637, USA; raptigh28@gmail.com

[7] Chemical Sciences and Engineering Division, Physical Sciences and Engineering Directorate, Argonne National Laboratory, Lemont, IL 60439, USA

[8] International Graduate Program of Molecular Science and Technology, National Taiwan University, Taipei 10617, Taiwan

[9] Molecular Science and Technology Program, Taiwan International Graduate Program, Academia Sinica, Taipei 10617, Taiwan

[10] College of Semiconductor Research, National Tsing Hua University, Hsinchu 30013, Taiwan

[11] Department of Materials Science and Engineering, National Tsing Hua University, Hsinchu 30013, Taiwan

* Correspondence: mario@phys.ntu.edu.tw

Fabrication of this structure requires the atomically precise assembly of transistor channels with molecular-scale thickness. We employed the method of Langmuir-Blodgett for producing large areas of functional monolayers for this purpose. Stearic acid mixed with hexanol in a ratio of 1:1 is used for this purpose because of its easy fabrication and well researched properties.

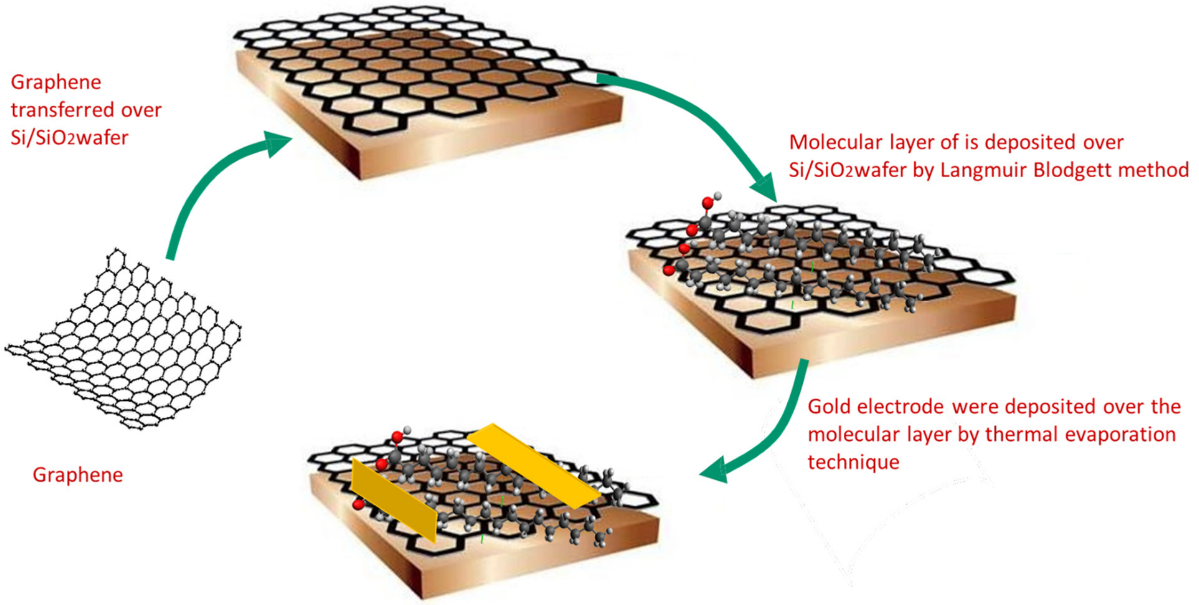

Figure S1: Fabrication steps for the device

In figure S2, the optical microscopy (OM) images, reveals two distinct regions separated by a layered boundary. One of these regions corresponds to the area where the Langmuir Blodgett (LB) film of stearic acid molecule over graphene has been deposited, while the other represents the substrate of the film. Figures 2(a) and 2(b) provide the quality of layer produce with a uniformity over the large area of the film.

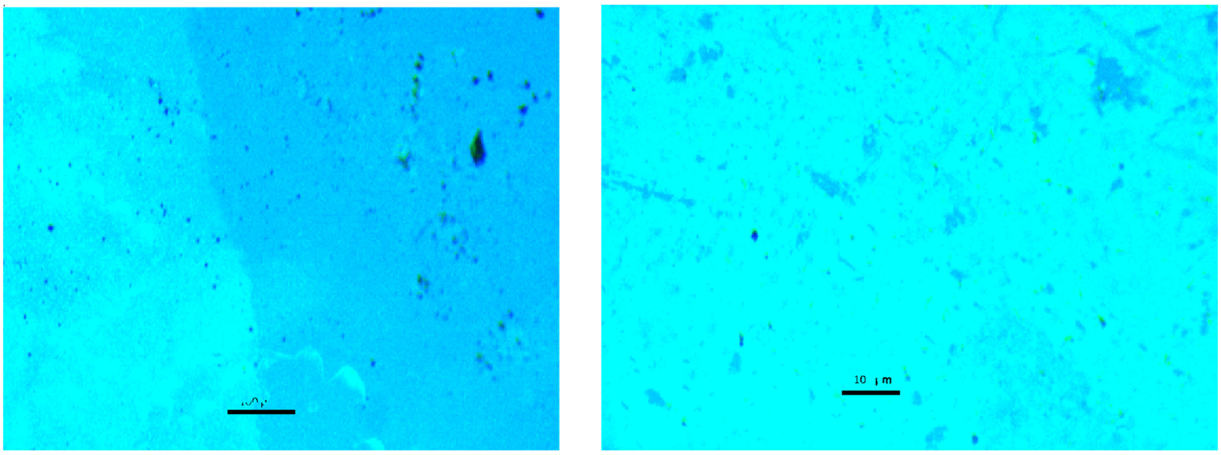

(a) (b)

Figure S2 Optical micrographs of stearic acid on Si/SiO_2 (a) interface between pristine SiO2/Si and single layer stearic acid, (b) micrograph of triple-layer stearic acid.

Raman spectroscopy is used to characterized graphene and the steric acid on the substrate. The results S3, obtained for the Raman spectra of graphene has G peak located at ~1580cm^{-1} and 2D peak at ~2700cm^{-1}. The Raman spectra of stearic shows the presence of peak between ~2800 cm^{-1} to ~2900cm^{-1}.

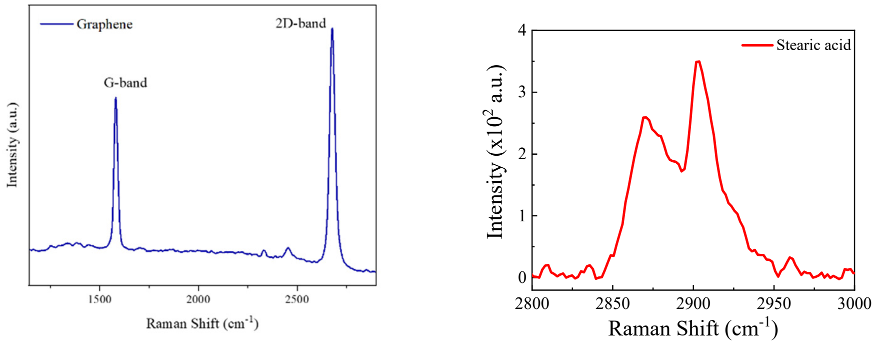

(a) (b)

Figure S3: (a)Raman spectra of graphene (b) Raman Spectra Langmuir-Blodgett film of stearic acid .

Time-domain thermoreflectance (TDTR) measurements were conducted by analyzing the change in reflectance of an 80nm Al film that was deposited on top of the stearic acid/graphene structure. For this purpose, a pump beam was utilized to heat the sample and the intensity of a reflected probe beam was analyzed through a lock-in technique. [1]

A model was utilized to extract the heat conductivity of the hybrid that considered the heat flow through multiple layers with different thickness, thermal conductivity, and heat capacity using tabulated data (see below) [2] The use of tabulated data permits a higher confidence toward the extracted thermal conductivity of the stearic acid/graphene hybrid, albeit at the cost of a decreased fitting quality.

	thickness	Thermal conductivity	Heat capacity
Aluminum	80nm	200 $Wm^{-1}K^{-1}$	2.44 $Jcm^{-3}K^{-1}$
Stearic acid/graphene	3nm	**Fit**	0.0005 $Jcm^{-3}K^{-1}$
SiO2	300nm	1.33 $Wm^{-1}K^{-1}$	1.48 $Jcm^{-3}K^{-1}$
Si	500nm	135 $Wm^{-1}K^{-1}$	1.62 $Jcm^{-3}K^{-1}$

1. Olaya, Daniel, Chien-Chih Tseng, Wen-Hao Chang, Wen-Pin Hsieh, Lain-Jong Li, Zhen-Yu Juang, and Yenny Hernandez. "Cross-Plane Thermoelectric Figure of Merit in Graphene-C60 Heterostructures at Room Temperature." *FlatChem* 14 (2019): 100089.

2. Cahill, D. G. "Analysis of Heat Flow in Layered Structures for Time-Domain Thermoreflectance." *Review of Scientific Instruments* 75, no. 12 (2004): 5119-22.
